# Smartphone-based molecularly imprinted sensors for rapid detection of thiamethoxam residues and applications

**DOI:** 10.1371/journal.pone.0258508

**Published:** 2021-11-08

**Authors:** Sihua Peng, Aqiang Wang, Yuyang Lian, Xi Zhang, Bei Zeng, Qiulin Chen, Heming Yang, Jinlei Li, Limin Li, Jianguo Dan, Jianjun Liao, Shihao Zhou

**Affiliations:** 1 Sanya Nanfan Research Institute of Hainan University, Hainan, China; 2 College of Plant Protection, Hainan University, Hainan, China; 3 College of Ecology and Environment, Hainan University, Hainan, China; 4 Key Laboratory of Germplasm Resources Biology of Tropical Special Ornamental Plants, College of Forestry, Hainan University, Hainan, China; Texas A&M University at Qatar, QATAR

## Abstract

In order to achieve rapid detection of thiamethoxam residues in mango, cowpea and water, this study modified the screen printed carbon electrode (SPCE) to make a specific molecular imprinting sensor (Thiamethoxam-MIP/Au/rGO/SPCE) for thiamethoxam. An integrated smartphone platform was also built for thiamethoxam residue analysis. The performance of the complete system was analyzed by cyclic voltammetry (CV) and differential pulse voltammetry (DPV). The system was then applied for the rapid determination of thiamethoxam residues in water, mango and cowpea samples. The results showed that the molecular sensor showed good linearity in the range 0.5–3.0 μmol/L of thiamethoxam. The detection limit of thiamethoxam was 0.5 μmol/L. Moreover, the sensor had good reproducibility and anti-interference performance. The average recovery rates of the pesticide residues in water, mango and cowpea samples were in the range of 90–110% with relative standard deviations < 5%. The rapid detection system for thiamethoxam residue constructed in this study was simple, reliable, reproducible and had strong anti-interference. It has broad application prospects in the field detection of thiamethoxam residue, and serves as a valuable reference for the further development of rapid detection technology of pesticide residues in the field of environment and food safety.

## Introduction

Thiamethoxam is a highly effective, low toxic and broad-spectrum neonicotinoid insecticide with a novel structure, which has good gastric toxicity, contact toxicity and endotoxic activity [1, 2]. It is mainly used for the effective control of Thripidae, Aphidoidea, *Trialeurodes vaporariorum* (Westwood) and other pests on fruit trees, vegetables and other crops [3, 4]. The safety of thiamethoxam is becoming a concern as its use becomes widespread and the doses applied become higher [5–7]. The detection of thiamethoxam residues in fruits, vegetables, soil and environmental water have been reported [8–10]. It is important therefore to develop a simple, rapid and reliable method for the detection of thiamethoxam residues in food and the environment, in order to protect consumers and life from its hazards.

Currently, the main methods for the detection of thiamethoxam residues are gas chromatography-mass spectrometry (GC-MS), high performance liquid chromatography (HPLC), liquid chromatography mass spectrometry (LC-MS) and other detection techniques [11–14]. These methods are sensitive, highly accurate, and reliable for quantitative analysis, but they require large instrumentation support and are expensive. They also have complex operating procedures that are time-consuming and require skilled operating techniques, limiting their application for the rapid detection of thiamethoxam residues. Compared to other techniques, electrochemical detection methods are simple to operate, low cost, and have fast response time, and therefore have great advantages for pesticide residue detection [15, 16].

Smartphones have become the most widely used mobile devices in recent years [17, 18]. Smartphones have become a research hotspot for portable testing devices in the field of biosensors because of their ability to receive and analyze data [19, 20]. Many researchers have used smartphones to combine various electrochemical methods such as cyclic voltammetry (CV), differential pulse voltammetry (DPV), and square wave voltammetry (SWV) to perform quantitative assays. For example, Ji et al. [21] developed an electrochemical detection system based on smart phones, using CV, DPV and other methods for rapid monitoring of levodopa. Another study used Au composite modified electrodes prepared by one-step co-electrodeposition as biosensors combined with a smartphone for the detection of circulating miR-21 biomarkers in saliva [22]. There are many more related studies [23, 24], but so far there are no smartphone-based sensors for the rapid detection of thiamethoxam concentrations.

In this study, we designed and built a smartphone-based electrochemical detection system by combining molecular imprinting technology with electrochemical detection technology. We prepared a portable molecular imprinting sensor for thiamethoxam (Thiamethoxam-MIP/Au/rGO/SPCE) by eluting a mixture which was made by modifying graphene, gold nanoparticles on SPCE and using chitosan (CS) as a functional matrix. Thiamethoxam was then used as a template molecule and glutaraldehyde as a cross-linking agent. The smartphone was integrated with a miniature electrochemical workstation, which consisted of a bluetooth module for connectivity. A specially designed android application was used to send commands and transfer data between the smartphone and the circuit board. This system was used to analyze the electrochemical behavior of the prepared sensors. Thereafter, the integrated analysis system was applied for the rapid detection of Thiamethoxam residues in mangoes, cowpeas and water.

## Materials and methods

### Main reagents

The main reagents used in this study were Thiamethoxam (TMX), chitosan (CS), graphene oxide (GO), sodium dihydrogen phosphate (NaH_2_PO_4_), dibasic sodium phosphate (Na_2_HPO_4_·12H_2_O), and glutaraldehyde (GA). They were purchased from Shanghai Aladdin Bio-Chem Technology Co., Ltd. Potassium chloride (KCl) was purchased from Sinopharm Chemical Reagent Co., Ltd. Acetic acid (CH_3_COOH) and potassium hexacyanoferrate(Ⅱ) Trihydrate (K_4_[Fe(CN)_6_]·3H_2_O) were purchased from Xilong Science Co. Red prussiate (K_3_[Fe(CN)_6_]) was purchased from Guangzhou Chemical Reagent Factory. Acetonitrile (C_2_H_3_N) was purchased from Shanghai Macklin Biochemical Co., Ltd. All the above reagents were analytically pure except Acetonitrile (C_2_H_3_N), which was chromatographically pure. The test water was ultrapure water.

### Major equipment

The main equipment used in this study were: an electrochemical workstation CHI660E (Shanghai Chenhua Instruments Co., Ltd.), an electronic analytical balance (Sartorius) (Germany), a table-top high-speed frozen centrifuge H3-18KR (Hangzhou Gengyu Instruments Co., Ltd.), a precision-type water purifier FST-111-TH100 (Puliflex), and electrodes of screen printing electrode SPCE E100.

### Preparation of molecularly-imprinted sensors

Following the method of Liang [25], but with slight modification, modified GO electrodes were prepared. Their parameters were then optimized by setting to a potential interval of -1.7 - +0.2 V, a rate of 0.05 V/S, and a number of turns of 32 seg. Cyclic voltammetry (CV) was then performed on a screen-printed electrode, SPCE. After drying for 24 h, chloroauric acid deposition solution (0.2% HAuCl_4_ solution configured with 0.5 mol/L H_2_SO_4_ as solvent) was added drop-wise and electrodeposited at -0.25 V for 180s using the constant potential method.

**Fig 1:**
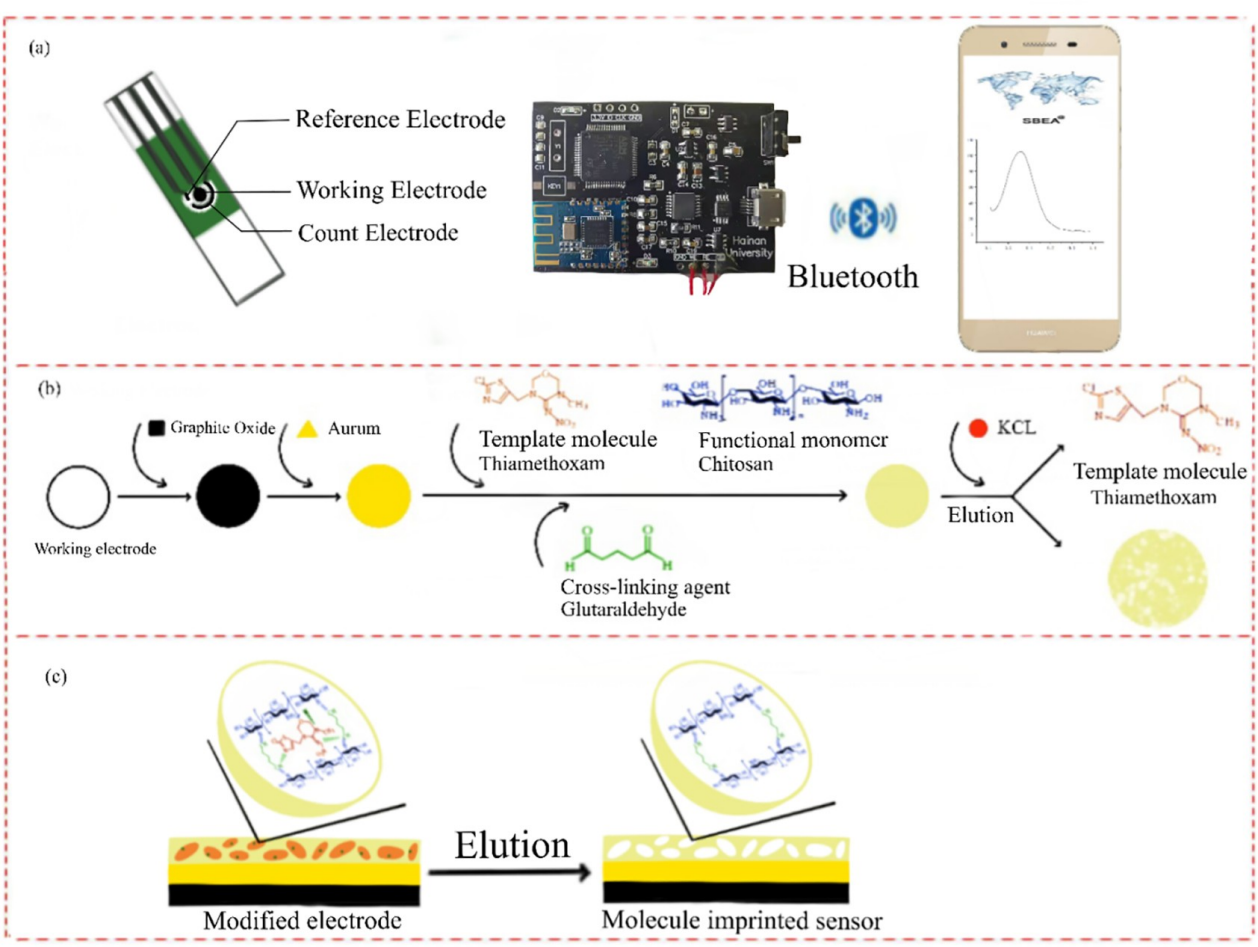
Schematic diagram of molecular imprinted sensor preparation. (a) Image of a mobile phone-based detection system including screen-printed electrodes, a miniature electrochemical workstation and a smartphone connected via bluetooth. (b) Schematic diagram of a molecularly-imprinted sensor preparation. (c) Internal structural changes of the elution step.

Following the method of Ming [26], but with slight modification, their use of imidacloprid pesticide was replaced with thiamethoxam in this study. Chitosan stock solution containing 1.0 mmol/L thiamethoxam (2 g of chitosan mixed with 2% glacial acetic acid and fixed to 50 mL, left overnight to become homogeneous and then weighed, to which 0.0128 g of thiamethoxam was added to configure a homogeneous solution) was dropped on the surface of the electrode that had been electrodeposited with chlorogoldeneic acid and polymerized at -1.0 V for 5 min. After the deposition was completed, the electrode was rinsed and dried and 5 μL of glutaraldehyde with a concentration of 0.1% was dropped on the surface of the electrode, dried, and then dried with ultrapure water to remove the excess glutaraldehyde. After that, 0.1 mol/L potassium chloride solution was dropped on the electrode and 100 seg was scanned at -0.4 - +0.8 V using cyclic voltammetry to elute the template molecules. The electrode obtained after completion was the molecularly-imprinted sensor with many thiamethoxam recognition sites on its surface (Fig 1). The electrode prepared by the preparation method without thiamethoxam in chitosan stock solution was used as a control (CK) (non-imprinted sensor).

### Electrochemical characterization of sensors

The electrochemical characterization of the sensor was performed by CV. A mixture of [Fe(CN)_6_]^3-/4-^ and KCl was added drop-wise to the prepared sensor (5.0 mmol/L [Fe(CN)_6_]^3-/4-^ mixture was prepared with 0.1 mol/L KCl as the solvent, similarly hereinafter). Subsequently, CV scans were performed for each electrode, and the potential scan range was: -0.2 - +0.6 V, at a rate of 0.05 V/S, and 10 seg of turns.

### Sensor performance testing methods

A mixture of [Fe(CN)_6_]^3-/4-^ and KCl was dropped on the sensor and scanned by differential pulse voltammetry (DPV), and the peak current of the sensor was recorded as *I*_0_ after the scan was completed. After drying, the sensors were immersed in phosphate buffered solution (PBS) (0.01 mol/L, pH 7.0) containing different concentrations of thiamethoxam for 30 min. After drying, a mixture containing [Fe(CN)_6_]^3-/4-^ and KCl was used as a probe and scanned by differential pulse voltammetry, and the peak current was recorded and noted as *I*. In this way, the prepared electrodes were tested using an electrochemical workstation and a homemade cell phone based portable sensing device. After the data were recorded, the inhibition rate (*I*%) of this sensor was calculated thiamethoxamusing the following equation:

$$I\%=\frac{I_{0}-I}{I_{0}}\times 100\%$$

### Repeatability testing

The same sensor was immersed in PBS solution containing 2 μmol/L thiamethoxam for 30 min, and then a mixture containing [Fe(CN)_6_]^3-/4-^ and KCl was used as the probe. It was scanned by DPV, and the peak current values were recorded. This operation was performed for 7 consecutive days. The relative standard deviation (RSD) values were then calculated.

### Anti-interference detection

An interfering solution made up of 1 μmol/L thiamethoxam solution as the stock solution, to which 5 μmol/L, 10 μmol/L and 20 μmol/L imidacloprid solutions were added was prepared. Imidacloprid was chosen as the interfering substance, because imidacloprid and thiamethoxam are typical representatives of the first and second generation neonicotinoid insecticides, respectively. They have relatively similar structures, and both are widely used for the control of the same type of pests. Therefore the residues of both chemicals are likely to appear in the same samples [27, 28] The same sensor was immersed in this solution for 30 min in a gradient. A mixture containing [Fe(CN)_6_]^3-/4-^ and KCl was then used as the probe. The peak current of the sensor was recorded by DPV The inhibition rate, as well as the difference between the inhibition rate of the interfering solution and that of the original solution was calculated.

### Pre-treatment of pesticide residue test samples

Water sample: a sample of water was taken from a river next to the tropical botanical garden in Danzhou City, Hainan Province, China (N19°30′43″, E109°30′1″) and was filtered in the laboratory using filter paper. Different amounts of thiamethoxam were added to the water sample to form a concentration gradient of 1μmol/L, 2μmol/L and 3μmol/L thiamethoxam for testing.

Mango samples: these were purchased from farmers’ markets. They were cleaned, dried, peeled, seeded and homogenized. A volume of 50mL of the juice was then dispensed in a centrifuge tube. The same volume of 1% acetic acid acetonitrile was then added. The mixture was then vortexed for 3min and then centrifuged at 8000r/min for 5min. The supernatant was taken and filtered. Different amounts of thiamethoxam were then added to the supernatant to obtain a concentration gradient of 1μmol/L, 2μmol/L and 3μmol/L thiamethoxam for testing.

Cowpea samples: these were purchased from farmers’ markets, washed and dried sufficiently. They were then chopped and polished for homogenization. The subsequent steps taken followed the same procedures as outlined for the preparation of the mango samples above.

### Residue detection of thiamethoxam in samples

The sensor was immersed in the testing samples of different concentrations of thiamethoxam for 30 min. After this, a mixture containing [Fe(CN)_6_]^3-/4-^ and KCl was used as the probe and scanned by DPV. The peak current from the scan was detected and recorded. Each sample was tested three times for each concentration. The recovery and the RSD values were then calculated.

### Data analysis

The statistical analyses in this paper are all expressed as relative standard deviations (RSD), which were calculated using Excel software with the following formula.


$$relative\;standard\;deviations\;(RSD)=\frac{arithmetic\;mean\;(X)}{standard\;deviation\;(SD)}$$

The arithmetic mean (X) of the data is a function of AVERAGE in Excel, and the standard deviation (SD) is a function of STDEV in Excel.

## Results

### Experimental parameter optimization

**Fig 2:**
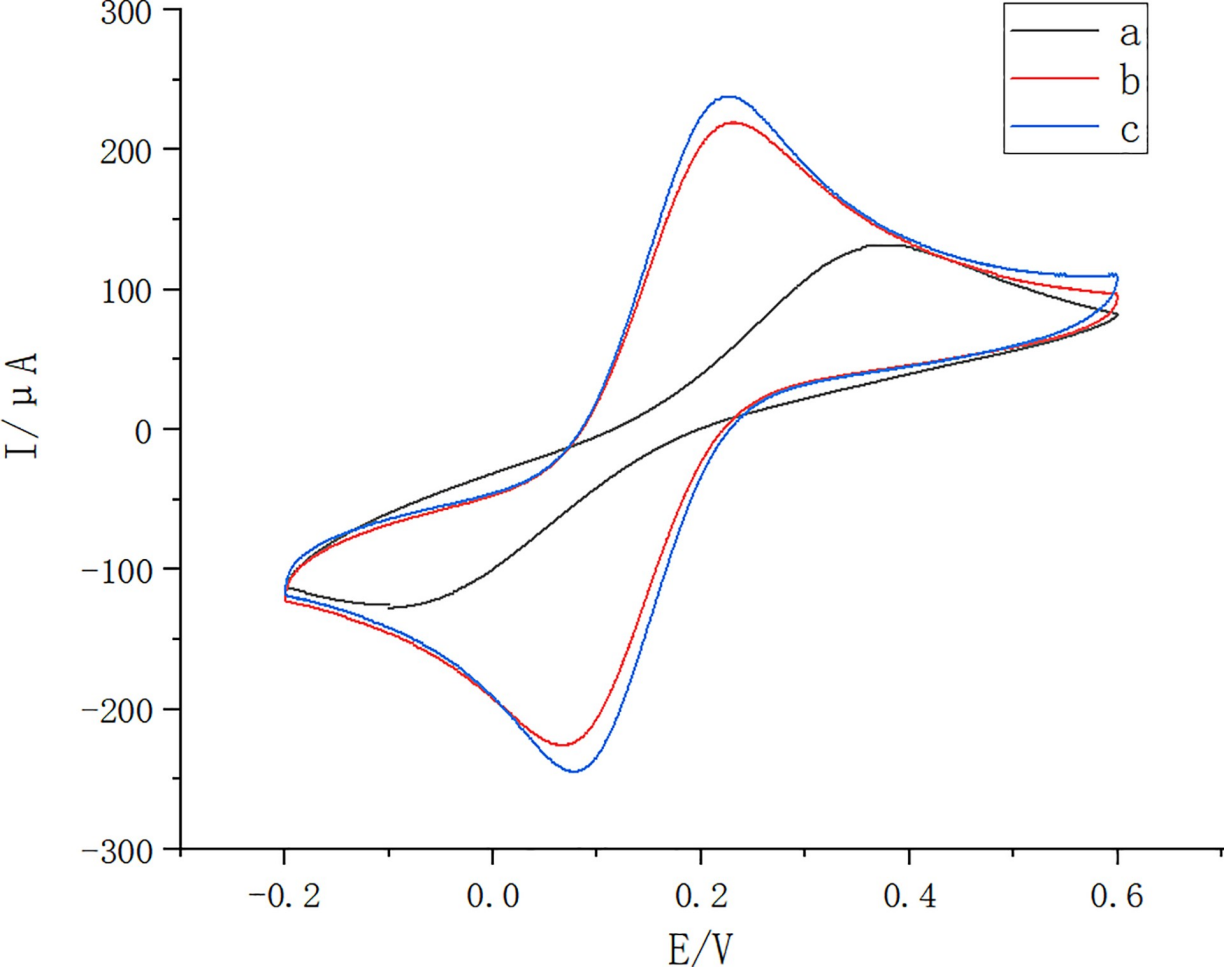
GO parameter optimization diagram. The original potassium ferricyanide curve without GO (black; a), the potassium ferricyanide curve after the original GO parameters (parameters of -1.6 - +0.6V) (red; b) and the potassium ferricyanide curve after the optimized GO parameters (parameters of -1.7 - +0.2V)(blue; c).

The modified GO electrode was optimized by adjusting the parameter from the potential range of -1.6 - +0.6V to -1.7 - +0.2V. The result showed that the peak cyclic voltammetric current after the adjustment was higher than that before the adjustment (Fig 2).

### Electrochemical characterization analysis results

#### Cyclic voltammetry

**Fig 3:**
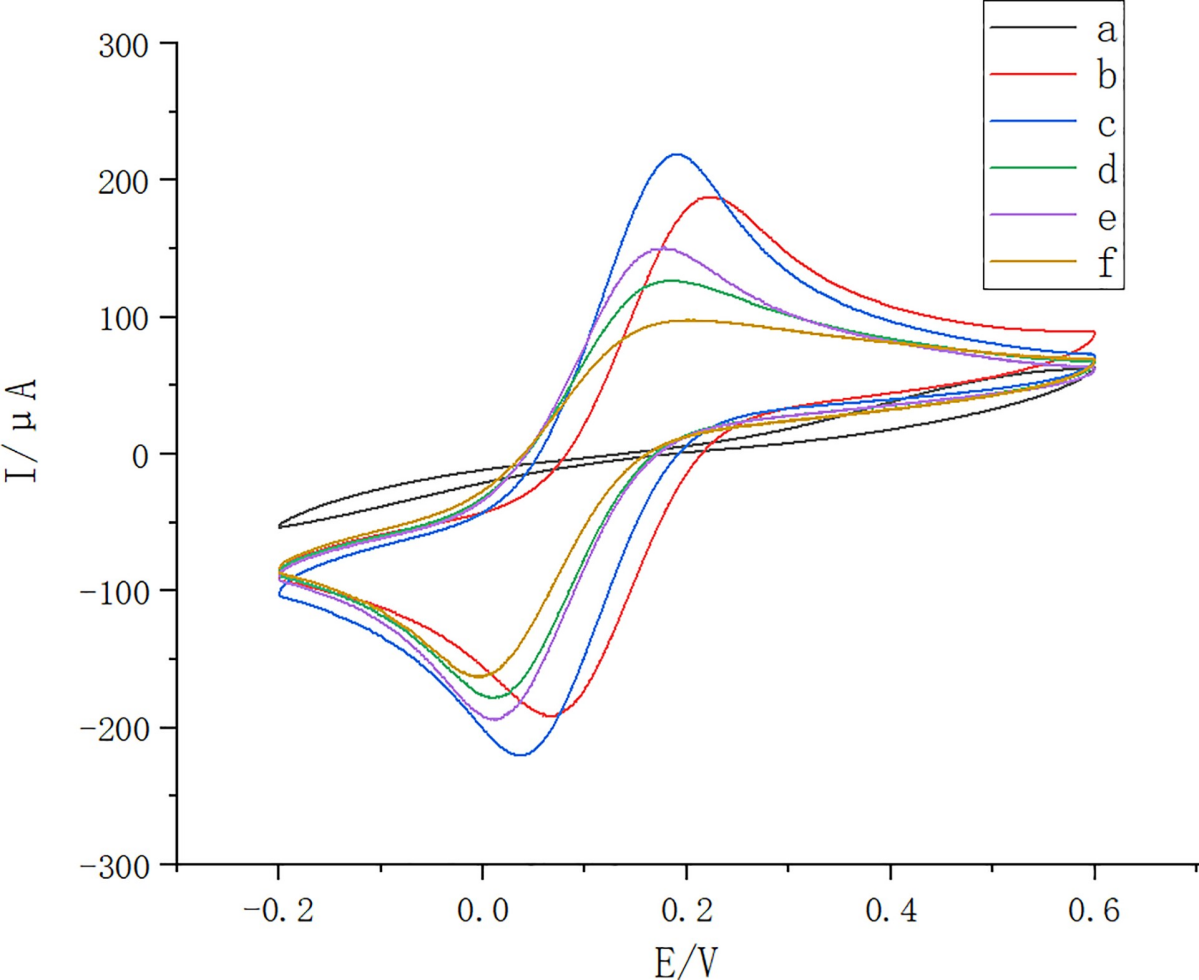
Cyclic Voltammetry (CV) curves for different electrodes. Bare electrode (bare SPCE)(black; a), GO modified post electrode (GO/SPCE)(red; b), GO and Au nanoparticle modified post electrode (Au/rGO/SPCE)(blue; c), chitosan polymerized glutaraldehyde cross-linked post electrode (CS/glutaraldehyde/SPCE)(green; d), molecularly imprinted sensor (Thiamethoxam-MIP/Au/rGO/SPCE)(purple; e), and non-molecularly imprinted sensor (N-Thiamethoxam-MIP/Au/rGO /SPCE) (CK)(yellow; f).

A portable molecularly-imprinted sensor (Thiamethoxam-MIP/Au/rGO/SPCE) for thiamethoxam was prepared by modifying SPCE. A designed android application was used to transmit data between the smart phone and the circuit board. CV analysis was performed on each electrode, with an electrochemical redox probe which was a mixture of [Fe(CN)_6_]^3-^/^4-^ and KCl. The results showed that the redox peaks of the above electrodes were all symmetrical (Fig 3). This indicates that the electrochemical reactions which occurred on the surface of the above electrodes were quasi-reversible. The peak current of the electrode after GO electroreduction was significantly higher than that of the bare electrode (Fig 3). This was due to the increased specific surface area and conductivity of the electrode after the modification of GO on the electrode surface. The electrode had the highest peak current after deposition of the chloroauric acid solution (Fig 3), which was due to the modification of the gold nanoparticles on the surface of the measured electrode. This increased the conductivity of the electrode and also the peak current. The peak current of the electrode (CS/glutaraldehyde/SPCE) after chitosan polymerization and glutaraldehyde crosslinking, was significantly reduced compared to GO and chloroauric acid (Fig 3). This was due to the covering of the original gold nanoparticles by chitosan, which reduced the conductivity of the electrode. The peak currents of the molecularly-imprinted sensors were much larger than those of the non-molecularly imprinted sensors. This suggested the imprinted sites on the surface of the imprinted membrane had good recognition properties for the thiamethoxam molecules (Fig 3).

### Exchanges impedance analysis (EIS)

**Fig 4:**
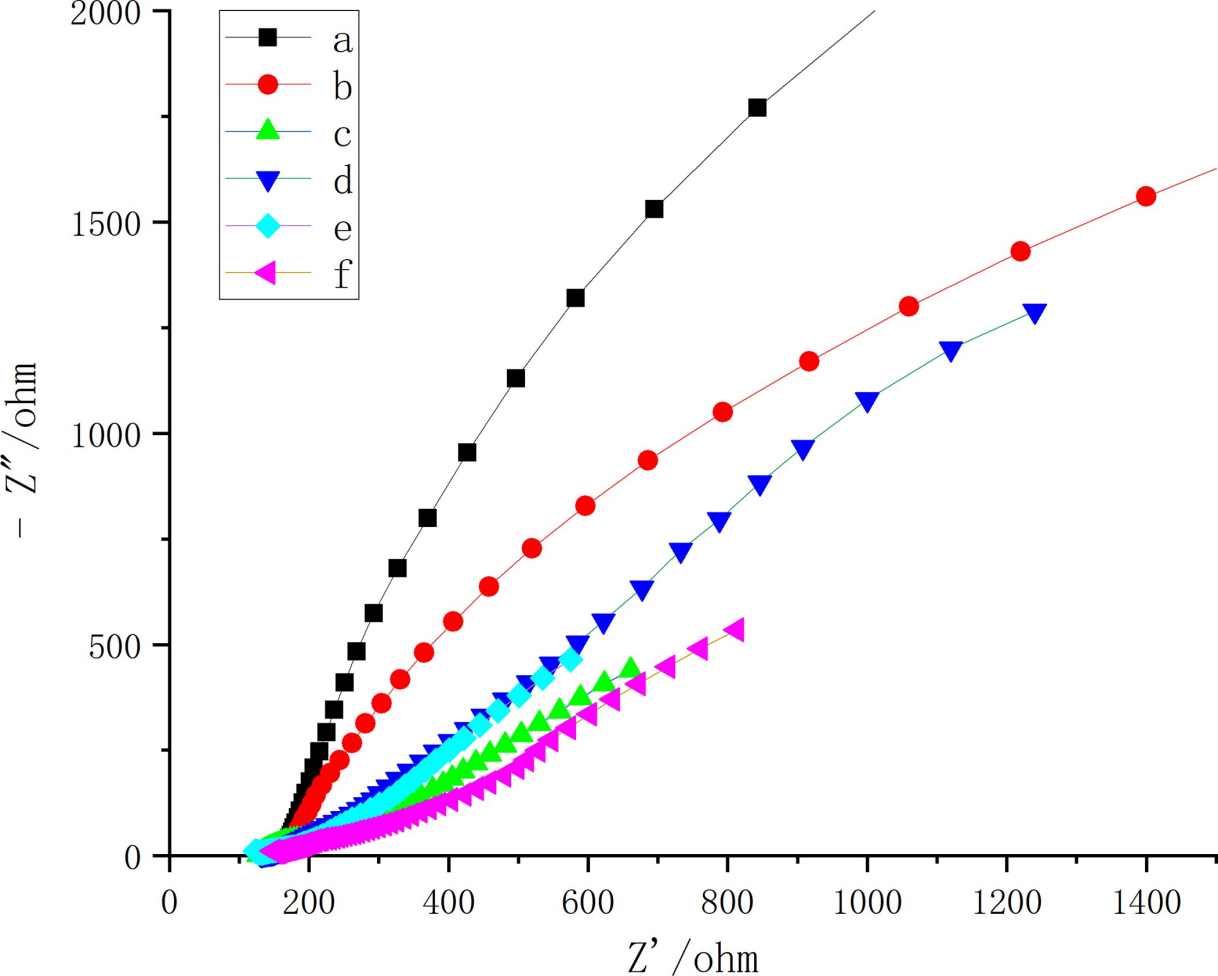
Exchanges impedance analysis (EIS) curves for different electrodes. Bare electrode (bare SPCE)(black; a), GO modified post electrode (GO/SPCE)(red; b), GO and Au nanoparticle modified post electrode (Au/SPCE)(green; c), Electrode after chitosan deposition (CS/SPCE)(blue; d), Glutaraldehyde crosslinked electrode (glutaraldehyde/SPCE)(wathet; e), molecularly imprinted sensor (Thiamethoxam-MIP/Au/rGO/SPCE)(purple; f).

Results showed that the bare electrode had the maximum resistance (Fig 4A), indicating that its conductivity was not good; after modification with graphene oxide and gold, the conductivity of the electrode was significantly enhanced (Fig 4B and 4C). Because chitosan was attached to the surface of gold, the conductivity of chitosan and glutaraldehyde modified electrode decreased (Fig 4D and 4E). After elution with potassium chloride, the template molecules fell off and exposed some holes, so the conductivity increased again (Fig 4F). The above changes showed the successful preparation of the electrode which specifically recognized the thiamethoxam template molecule.

### Morphological characterization of each electrode

**Fig 5:**
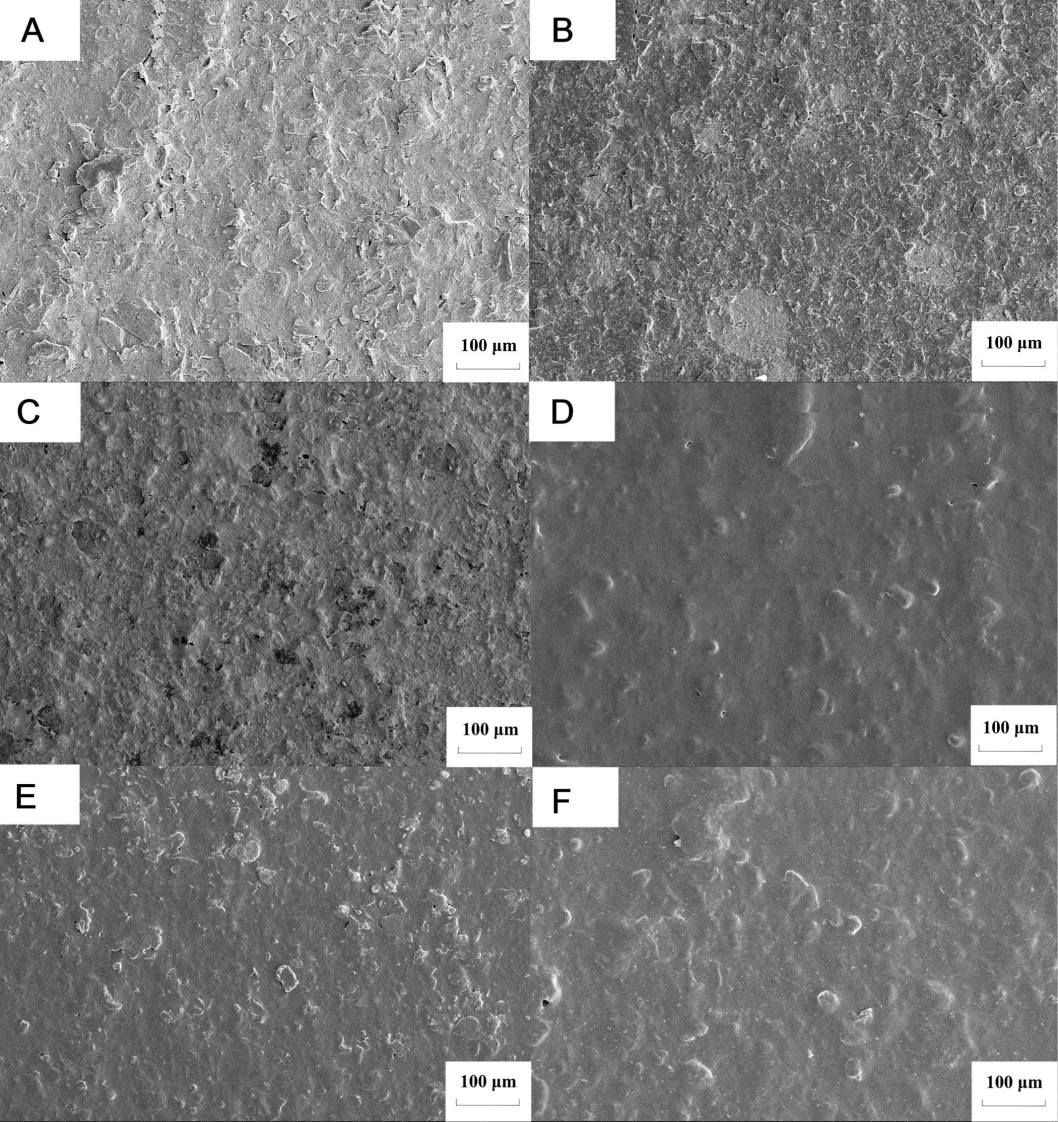
Electron microscopic analysis of each electrode. (A) The bare electrode surface of screen printed carbon electrode. (B) The electrode surface after graphene oxide (Go) modification (C) The electrode surface after chloroauric acid modification. (D)The electrode surface after chitosan deposition.(E) The electrode surface after glutaraldehyde crosslinking. (F) The electrode surface of molecularly imprinted sensor after elution.

Results showed that the bare electrode surface of screen printed carbon electrode was rough and loose (Fig 5A). After modification, the surface was dense and the gap became small (Fig 5B), indicating that graphene was successfully attached to the bare electrode surface. Compared to B, surface C had an obvious particle feeling, indicating that the gold particles had been repaired on the electrode surface. Surface D was smooth and had an obvious film feeling, indicating that chitosan had been successfully deposited. After crosslinking with glutaraldehyde, the electrode surface showed some block bulges, indicating that the structure had changed and the crosslinking was successful (Fig 5E). After elution with potassium chloride, the bulges on the surface of F were significantly reduced and the surface was smoother, indicating that the template molecules were eluted. The above pictures show that cyromazine MIP / Au / rGO had been successfully modified on to the electrode surface.

### Differential pulse voltammetry (DPV) results of the sensor on template molecules

**Fig 6:**
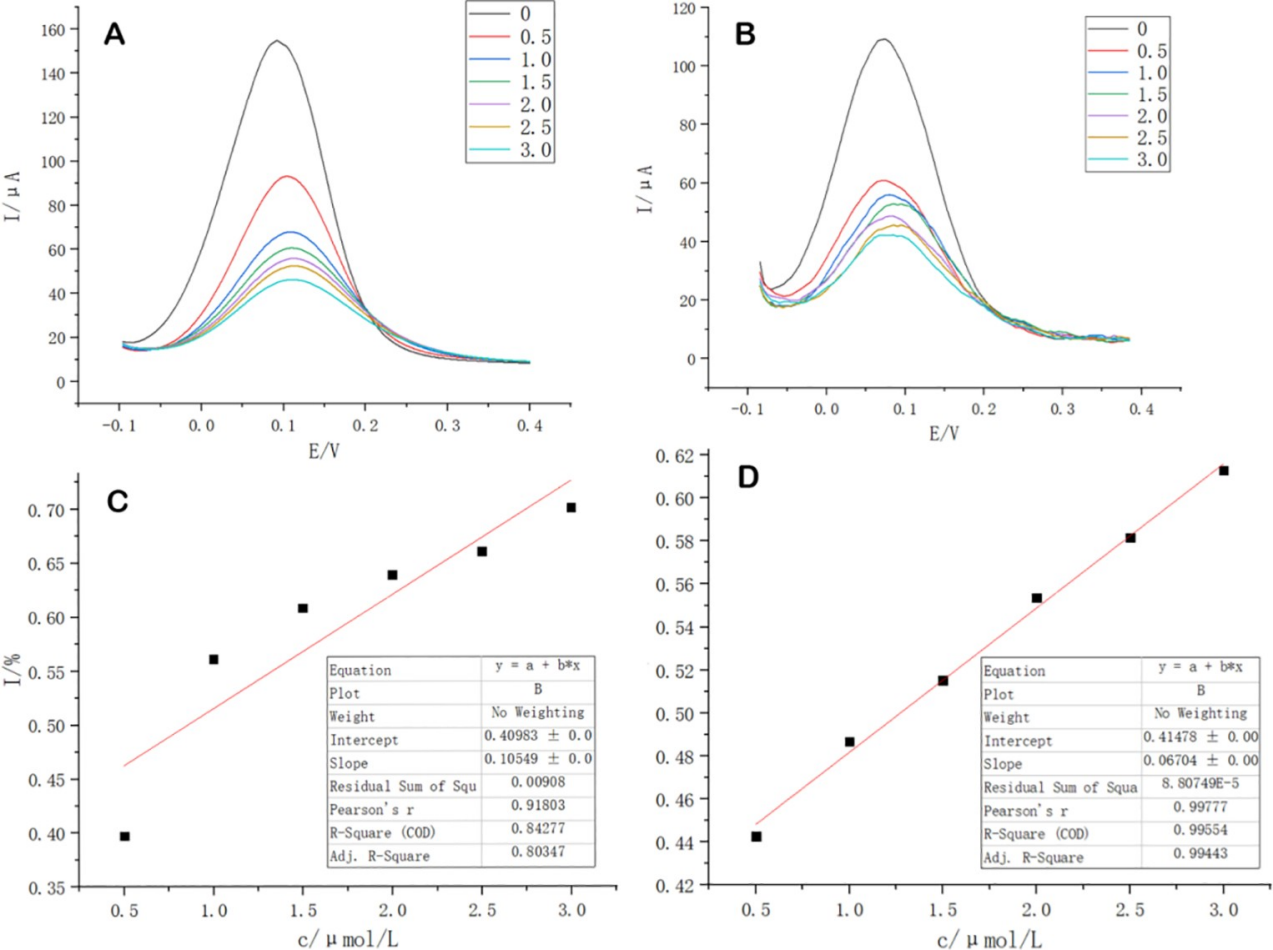
Comparison of commercial electrochemical workstations and portable sensors for smartphone platforms. (A) DPV curves of molecular imprinting sensor on electrochemical workstation for different concentrations of thiamethoxam. (B) DPV curves of portable molecular imprinting sensor based on cell phone for different concentrations of thiamethoxam. (C) Inhibition rate curves of molecular imprinting sensor on electrochemical workstation for different concentrations of thiamethoxam. (D) Inhibition rate curves of portable molecular imprinting sensor based on cell phone for different concentrations of thiamethoxam. Note: 0, 0.5, 1.0, 1.5, 2.0, 2.5, 3.0 are different concentrations of thiamethoxam, where the concentration units are μmol/L.

Differential pulse analysis of the prepared molecularly-imprinted sensors was performed on an electrochemical workstation and a portable sensor based on a smartphone platform. When the concentration of thiamethoxam changed, the response currents of both the electrochemical workstation and the mobile phone-based portable sensor changed accordingly thiamethoxam. Also when the thiamethoxam concentration increased, the corresponding response currents decreased accordingly (Fig 6A, 6B). This indicated the good performance of both the electrochemical sensor and the homemade portable sensor for the detection of thiamethoxam. Thiamethoxam concentration higher than 0.5 μmol/L had a good response which was exhibited accordingly by the corresponding peak current. The reason for this performance was that the target molecules present in the solution were located around the molecularly-imprinted sensor and their occupation of the recognition sites on the electrode surface ensured the exhibition of the good peak currents. There was a strong linear relationship between the response current and the pesticide concentration within a certain concentration range. The linear fit of the detection results of the mobile phone-based portable sensor was higher than that of the electrochemical workstation (Fig 6C, 6D).

### Repeatability analysis of sensors

**Fig 7:**
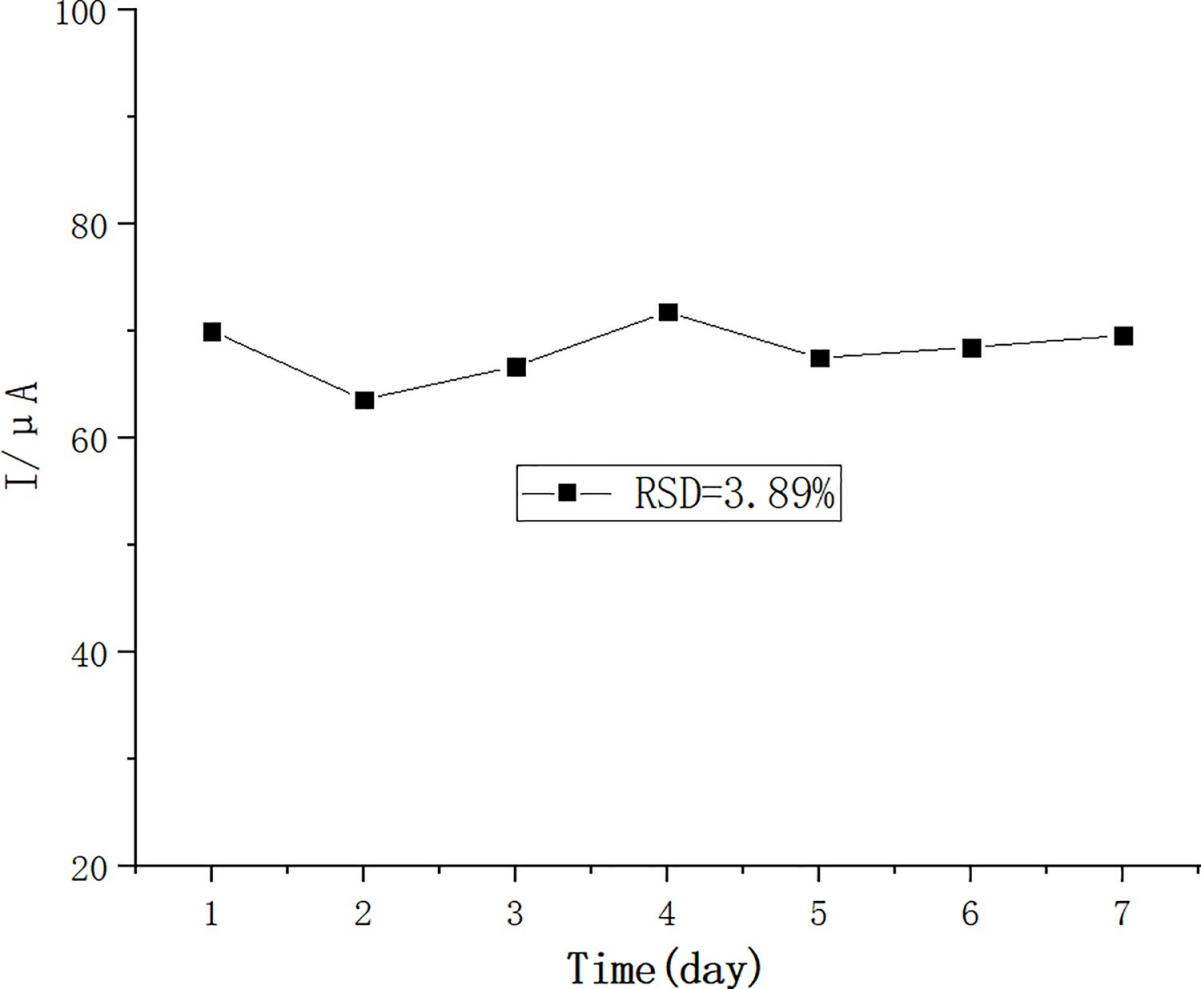
Results of the repeatability analysis of the sensor.

The results from this experiment showed that the RSD value for 7 consecutive days was 3.89%. There was no significant decrease in the peak current after 7 d (Fig 7), indicating that the prepared sensor had good repetitive stability.

### Anti-interference analysis of sensors

**Fig 8:**
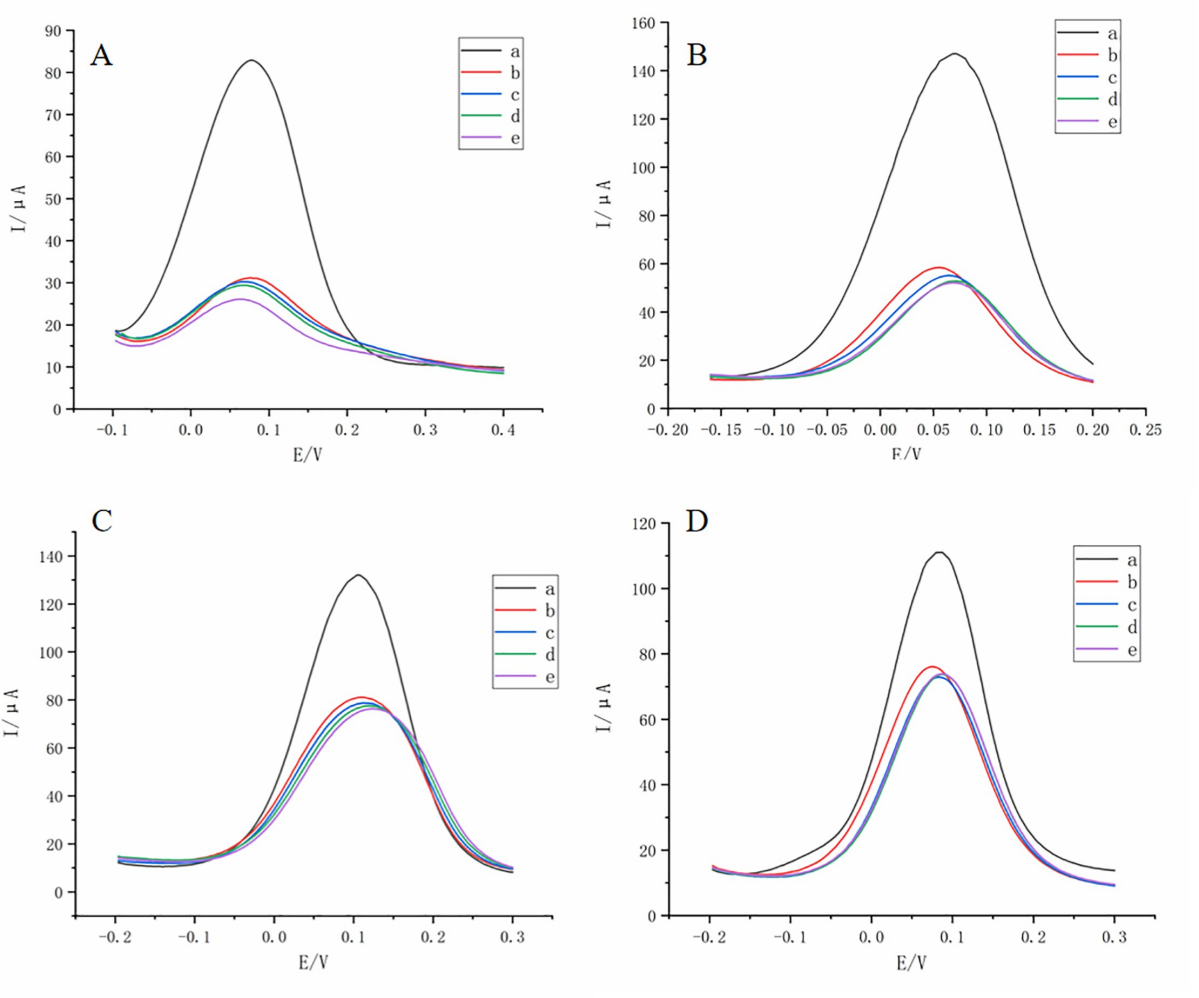
Anti-interference DPV analysis. (A) DPV curve of imidacloprid as interfering substance. (B) DPV curve of acetamiprid as interfering substance. (C) DPV curve of dinotefuran as interfering substance. (D) DPV curve of clothianidin as interfering substance. For the curves in the above four figures: Original DPV curve (black;a), DPV curve after immersion in 1μmol/L thiamethoxam solution (stock solution)(red; b), DPV curve after immersion in 1μmol/L thiamethoxam mixed with 5μmol/L Interfering substance (blue; c), DPV curve after immersion in 1μmol/L thiamethoxam mixed with 10μmol/L Interfering substance (green; d), and DPV curve after immersion in 1μmol/L thiamethoxam mixed with 20μ mol/L Interfering substance (purple; e).

The results of four interfering substances showed that except the original DPV, the DPV curve was distributed in clusters. The DPV curves obtained from the 1 μmol/L thiamethoxam solution were similar to those obtained by adding 5 μmol/L, 10 μmol/L, and 20 μmol/L interfering substance solutions to 1 μmol/L thiamethoxam solution (Fig 8).The differences in inhibition rates between the thiamethoxam solutions containing 5 μmol/L and 10 μmol/L interfering substance were all less than 5%, while the differences in inhibition rates between the thiamethoxam solutions containing 20 μmol/L imidacloprid and the original solution were 6.53%, which were less than 10%. The others were within 5% (Table 1). These results indicated that the prepared thiamethoxam-specific molecular imprinting sensor had good interference resistance.

**Table 1:** Results of interference immunity analysis of sensors.

Interfering substance	concentration	Difference between inhibition rate and stock solution
imidacloprid	5	1.28%
	10	2.33%
	20	6.53%
acetamiprid	5	2.27%
	10	3.83%
	20	4.29%
clothianidin	5	2.88%
	10	2.16%
	20	1.98%
dinotefuran	5	1.74%
	10	2.73%
	20	3.64%

### Analysis of thiamethoxam residues in actual samples

The spiked recovery rates of thiamethoxam from the water, mango and cowpea samples ranged from 97.68% to 106.54%, 98.35% to 100.48% and 100.32% to 104.76%, respectively; and the relative standard deviations were all <4.45% (Table 2). The results were in accordance with the national food physical and chemical detection standard (gb-t-27404-2008—laboratory quality control standard—food physical and chemical detection), indicating that the prepared molecular imprinted sensor can meet the requirements of rapid detection of thiamethoxam in actual samples.

**Table 2:** Analytical results of thiamethoxam residues in actual samples.

Samples	Added (μmol/L)	Found (μmol/L)	Recovery (n = 3)	RSD (n = 3)
Water	1	1.07	106.54%	3.39%
	2	1.96	97.93%	4.45%
	3	2.93	97.68%	2.74%
Mango	1	0.98	98.35%	1.84%
	2	2.01	100.48%	4.39%
	3	2.98	99.45%	3.54%
Cowpea	1	1.05	104.76%	1.26%
	2	2.01	100.38%	3.79%
	3	3.01	100.32%	1.22%

## Discussion

In recent years, rapid detection techniques for pesticide residues have been developed. Nowadays more researches are being conducted on enzyme inhibition techniques, enzyme immunoassay techniques, and biosensor techniques [29, 30]. Electrochemical biosensing technology does not only have good accuracy and sensitivity, but also has the advantages of easy operation, low price and strong anti-interference ability. For example, the electrochemical biosensor based on screen printing technology can realize the industrial production of large quantities, simple preparation, easy portability, cost reduction, fast and accurate measurement. It can also avoid the memory effect of conventional solid electrodes and the complicated and lengthy pretreatment process [31, 32]. However, most of these techniques are currently applied for the detection of organophosphorus pesticides, carbamate pesticides and herbicides. The detection of nicotine pesticides is still in its infancy. In this study, we designed a cell phone software for an electrochemical detection system based on screen-printing technology and electrochemical biosensor technology. This system together with a home-made device was used to modify a screen-printed electrode and also to prepare a sensor which showed excellent performance. The system was then used to analyse thiamethoxam residues in samples.thiamethoxam The results showed that the detection limit of thiamethoxam was 0.5 μmol/L, and the recovery rates from the actual samples were in the range of 90–110%. The relative standard deviations (RSD) were less than 5%. Mohamed Khairy [33] *et al*. used nickel oxide nanosheets (NPs) modified with screen-printed electrodes to build a nanoenzyme sensor to test parathion pesticides in urine and tomato juice samples. The recovery rates ranged from 90% to 110% and standard deviations around 5%, similar to the results of this experiment. Flavio della Pelle [34] and others also constructed electrochemical biosensors based on screen printing technology to detect the recoveries of carbamate pesticides in soft wheat, hard wheat and maize. They reported a recovery rate which ranged from 78% to 102%, which was lower than that recorded from this experiment. The good results obtained from all experiments conducted in this study showed that the system has a broad developmental prospect.

Comparison of the performance results between the homemade portable sensor and the electrochemical workstation in this study showed that the linear fit of the detection results of the mobile phone-based portable sensor was higher than that of the electrochemical workstation. This indicated that this portable sensor was more accurate and has the prospect of vigorous development. However, the DPV curve of the portable sensor was not as rounded as the electrochemical workstation, and the curve had twists and turns. This may be due to the poor performance of the electrode in this experiment. The electrode used in this experiment was a common screen-printed carbon electrode. Conductive carbon paste is the most commonly used material for oil printing due to its inexpensiveness, easy modification and chemically inert characteristics. The type of conductive phase (carbon powder) in the conductive carbon paste, particle size and conductive film resistance can have an effect on the performance [35].However, the electrode performance can be improved by adding graphene to the carbon paste or using other materials and other methods [36, 37]. The performance can also be improved by adopting the procedures of Zhao *et al*. [38] to improve the blot ratio, cross-linker and solvent selection.

Studies on enzyme sensors, immunosensors, nucleic acid sensors, and molecularly imprinted sensors for pesticides have been conducted [39–41]. For example, cholinesterases have been reported to be involved in detoxification processes due to their ability to scavenge organophosphates and carbamates. Following this, electrochemical cholinesterase sensors for pesticide detection have received increasing attention [42].The detection system used in this paper used a combination of molecular blotting techniques for the detection of thiamethoxam residues. In the future, this system can be coupled with cholinesterase and other categories of sensors for the rapid detection of organophosphorus and carbamate pesticide residues. This will expand the detection categories and applications of this system.
